# Prognostic impact of PD-L1 expression in correlation with neutrophil-to-lymphocyte ratio in squamous cell carcinoma of the lung

**DOI:** 10.1038/s41598-019-57321-x

**Published:** 2020-01-27

**Authors:** Yuko Tashima, Taiji Kuwata, Kazue Yoneda, Ayako Hirai, Masataka Mori, Masatoshi Kanayama, Naoko Imanishi, Koji Kuroda, Yoshinobu Ichiki, Fumihiro Tanaka

**Affiliations:** Second Department of Surgery (Chest Surgery), University of Occupational and Environmental Health, Kitakyushu, Japan

**Keywords:** Non-small-cell lung cancer, Prognostic markers

## Abstract

The prognostic impact of tumoral programmed death-ligand 1 (PD-L1) expression in correlation with neutrophil-to-lymphocyte ratio (NLR) was retrospectively assessed in 83 patients with completely resected stage I squamous cell carcinoma of the lung, as PD-L1 is a potent regulator of cancer immunity and NLR is a potential surrogate of immune status. Forty-three patients (51.8%) had tumor with positive PD-L1 expression. There was no significant correlation between PD-L1 expression and NLR. PD-L1-positivity failed to provide a significant prognostic impact (overall survival [OS] rate at 5 years, 53.0% in PD-L1-positive patients versus 70.1% in PD-L1-negative patients; *P* = 0.117). Among NLR-low (<2.2) patients, however, PD-L1-positivity was significantly correlated with a poor prognosis (OS rate at 5 years, 46.1% versus 86.0%; *P* = 0.020). In contrast, among NLR-high (≥2.2) patients, PD-L1-positivity provided no prognostic impact (*P* = 0.680). When NLR status and tumoral PD-L1 status were combined, “NLR-low and PD-L1-negative” was a significant and independent factor to predict a favorable recurrence-free survival (hazard ratio, 0.237 [95% confidence interval, 0.083 to 0.674]; *P* = 0.007) and OS (hazard ratio, 0.260 [0.091 to 0.745]; *P* = 0.012). These results suggest the prognostic impact of tumoral PD-L1 expression might be influenced by the status of NLR.

## Introduction

Squamous cell carcinoma is a pathologic subtype of primary lung cancer that is the leading cause of cancer deaths worldwide^1^. Although surgery is recommended as a standard care of treatment for early-stage squamous cell carcinoma, the postoperative prognosis remains poor despite recent improvement of adjuvant chemotherapy following surgery^2,3^. Accordingly, development and establishment of novel prognostic and predictive markers is essential to improve the postoperative survival^3^.

Programmed cell death protein 1 (PD-1) is an immune checkpoint molecule that negatively regulates immune system^4^. Among two ligands of PD-1 (PD-L1 and PD-L2), PD-L1 is predominantly expressed on tumor cells (TCs) in a wide variety of malignant tumors such as non-small cell lung cancer (NSCLC). PD-L1 binds to PD-1 on activated cytotoxic T lymphocytes (CTLs), which leads to down-regulation of immune attack by CTLs and survival of TCs^4,5^. Accordingly, blockade of PD-1/PD-L1 axis can be a promising strategy to kill TCs with strong expression of PD-L1. In fact, tumoral PD-L1 expression status has been approved for clinical use as a biomarker to predict the efficacy of pembrolizumab, an anti-PD-1 antibody, in NSCLC^4–7^.

Tumoral PD-L1 expression status may also provide a prognostic information, as PD-L1 plays critical roles in development and progression of malignant tumors through immune evasion of TCs. However, the prognostic significant of tumoral PD-L1 status remains controversial, as inconsistent results have been reported in several retrospective clinical studies^8,9^. One possible reason for such conflict results is that the prognostic impact of tumoral PD-L1 status can be influenced by the status of cancer immune activity and by several stimulatory and/or inhibitory factors associated with cancer immunity other than PD-L1^5,7,9^. The neutrophil-to-lymphocyte ratio (NLR), which is easily calculated by dividing the number of neutrophils by number of lymphocytes, is a potential surrogate of systemic inflammation. Many clinical studies revealed that high NLR was associated with a poor prognosis in NSCLC^10,11^. Recently, the NLR has merged as an indicator of immune status, as it is associated with the survival benefit of PD-1/PD-L1 inhibitors^12–14^. Here, we examined the prognostic impact of tumoral PD-L1 expression status in correlation with NLR in early-stage lung squamous cell carcinoma.

## Results

### Distribution of NLR and cut-off value for prognostic analyses

**Figure 1:**
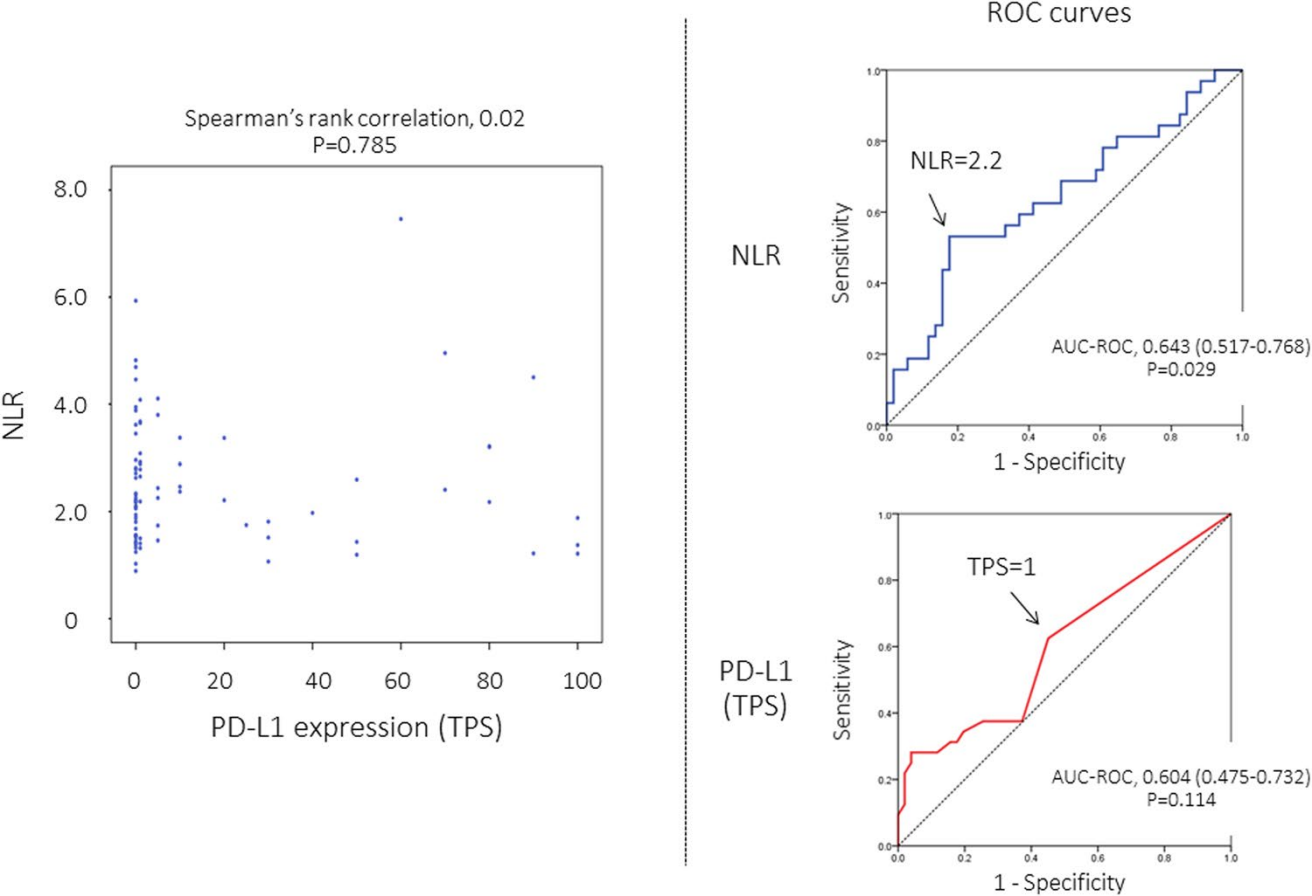
Distribution of neutrophil-to-lymphocyte ratio (NLR) and tumor proportion score (TPS) for tumoral PD-L1 expression (left). Receiver operating characteristics (ROC) curves to examine diagnostic performance of NLR (right upper) and TPS (right lower) for prediction of death from any cause. AUC-ROC, area under ROC curve.

The NLR value of each case was indicated in Fig. 1. The receiver operating characteristic (ROC) curve analysis showed that NLR provided a significant but modest diagnostic performance to predict death (are under ROC curve [AUC-ROC], 0.643; *P* = 0.029) (Fig. 1). Based on the ROC curve, the median value (2.2) was employed as the cut-off value to classify each patient into NLR-high (NLR, 2.2 or higher) or NLR-low (NLR, less than 2.2) patient in further survival analyses (Fig. 1).

### Recurrence-free survival (RFS) and overall survival (OS) according to NLR status

**Figure 2:**
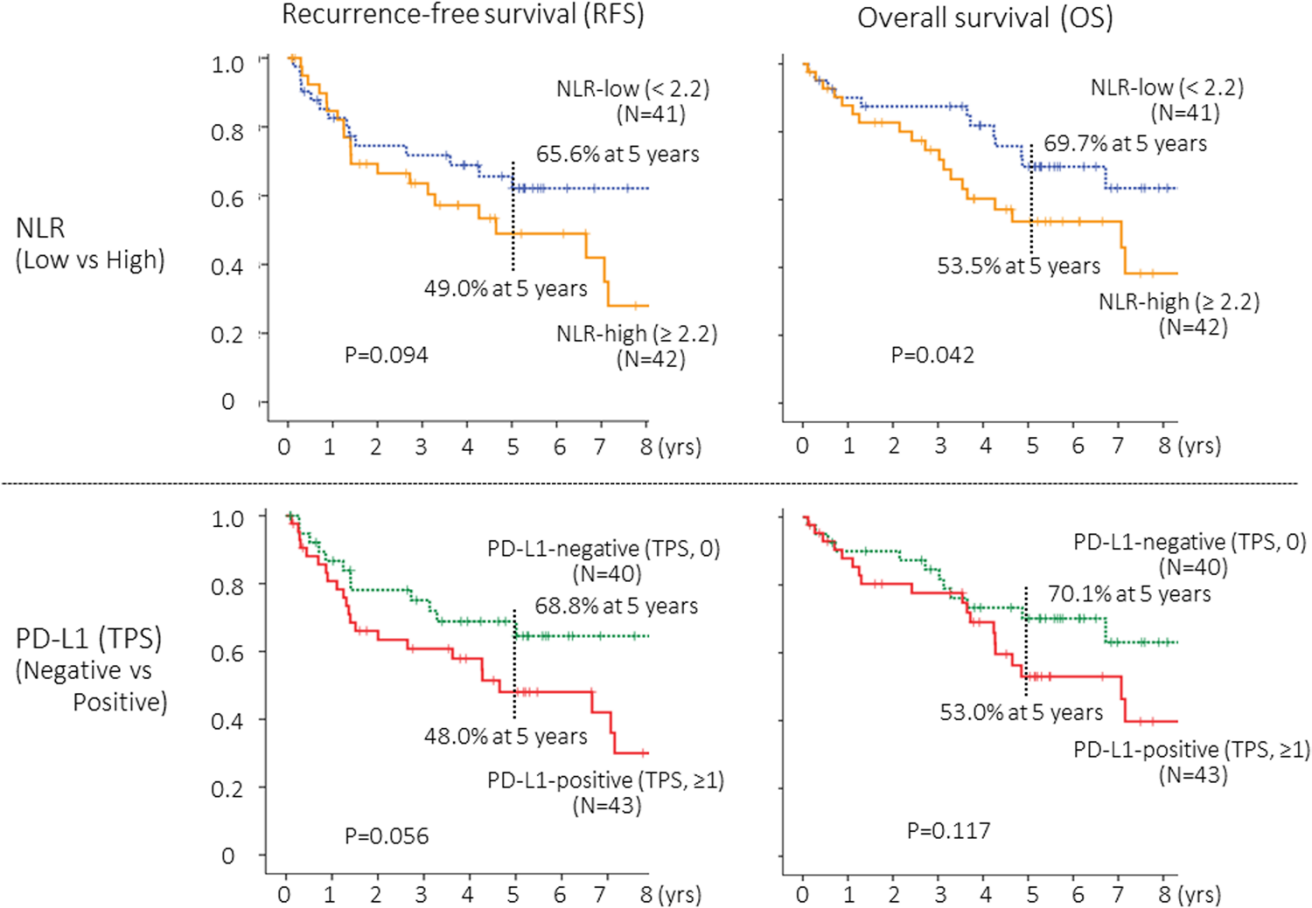
Recurrence-free survival (PFS) and overall survival (OS) curves according to neutrophil-to-lymphocyte ratio (NLR) and tumor proportion score (TPS) for tumoral PD-L1 expression status. The cut-off value for NLR and TPS were 2.2 and 1, respectively.

The NLR provided a significant but modest prognostic impact for overall survival (OS) (*P* = 0.042), and its prognostic impact did not reach a statistical significance for recurrence-free survival (RFS) (*P* = 0.094) (Fig. 2).

### PD-L1 expression (tumor proportion score, TPS) in correlation with other patient characteristics

The distribution of TPS was indicated in Fig. 1, and there was no significant correlation between NLR and TPS. PD-L1 expression status in correlation with several patient characteristics were indicated in Table 1. The Brinkman index was significantly higher in PD-L1-positive patients at higher cut-off values of TPS (TPS, 10 and 50). There was no significant difference in any other characteristics according to PD-L1 positivity.

**Table 1:** PD-L1 expression (TPS) in correlation with patient characteristics.

TPS	≥1%	0	P-	≥5%	0–4	P-	≥10%	0–9	P-	≥50%	0–4	9
PD-L1 status	positive	negative		positive	negative		positive	negative		positive	negative	P-
Age, median (years)	75.0	73.0	0.468	73.0	74.0	0.442	74.0	73.5	0.901	71.0	73.9	0.221
**Sex**												
female	6 (14.0)	7 (17.5)	0.766	6 (19.4)	7 (13.5))	0.539	4 (16.0)	9 (15.5)	1.000	2 (14.3)	11 (15.9)	1.000
male	37(86.0)	33 (82.5)		25 (80.6)	45 (86.5)		21 (84.0)	49 (84.5)		12 (17.1)	58 (84.1)	
**Smoking status**												
never	0 (0.0)	1 (2.5)		0 (0.0)	1 (1.9)		0 (0.0)	1 (1.7)		0 (0.0)	1 (1.4)	
former	18 (41.9)	21 (52.5)	0.322	15 (48.4)	24 (46.2)	0.735	10 (40.0)	29 (50.0)	0.531	5 (35.7)	34 (49.3)	0.559
current	25 (58.1)	18 (41.9)		16 (51.6)	27 (51.9)		15 (60.0)	28 (48.3)		9 (64.3)	34 (49.3)	
Brinkman index, median (pack-year)	56.0	53.0	0.369	56.0	53.8	0.197	60.0	51.0	0.033	61.3	52.0	0.040
**Cell differentiation**												
well	5 (11.6)	6 (15.0)		3 (9.7)	8 (15.4)		3 (12.0)	8 (13.8)		2 (18.2)	9 (13.0)	
moderately	29 (67.4)	25 (62.5)	0.870	22 (71.0)	32 (61.5)	0.648	18 (72.0)	36 (62.1)	0.657	8 (57.1)	46 (66.7)	0.762
poorly	9 (20.9)	9 (22.5)		6 (19.4)	12 (23.1)		4 (16.0)	14 (24.1)		4 (28.6)	14 (20.3)	
**Pathologic stage**												
IA	22 (51.2)	19 (47.5)	0.827	15 (48.4)	26 (50.0)	1.000	13 (52.0)	28 (48.3)	0.814	7 (50.0)	34 (49.3)	1.000
IB	21 (48.8)	21 (52.5)		16 (51.6)	26 (50.0)		12 (48.0)	30 (51.7)		7 (50.0)	35 (50.7)	
**Mode of lung resection**												
Sub-lobar resection	9 (20.9)	9 (22.5)	1.000	8 (25.8)	10 (19.2)	0.584	6 (24.0)	12 (20.7)	0.776	3 (21.4)	15 (21.7)	1.000
Lobectomy	34 (79.1)	31 (77.5)		23 (74.2)	42 (80.8)		19 (76.0)	46 (79.3)		11 (78.6)	54 (78.3)	
**Adjuvant chemotherapy**												
Performed	4 (9.3)	7 (17.5)	0.340	2 (6.5)	9 (17.3)	0.197	1 (4.0)	10 (17.2)	0.160	0 (0.0)	11 (15.9)	0.197
NLR, median	2.41	2.14	0.397	2.26	2.20	0.932	2.21	2.21	0.812	2.29	2.21	0.942
low	18 (41.9)	23 (57.5)	0.190	14 (45.2)	27 (51.9)	0.651	12 (48.0)	29 (50.0)	1.000	7 (50.0)	34 (49.3)	1.000
high	25 (59.5)	17 (42.5)		17 (54.8)	25 (48.1)		13 (52.0)	29 (50.0)		7 (50.0)	35 (50.7)	

NLR, neutrophil to lymphocyte ratio; Data represented as absolute counts (%) or median.

### Prognostic impact of PD-L1 expression status

The ROC curve analysis failed to show a significant diagnostic performance of TPS to death (AUC-ROC, 0.604; *P* = 0.114) (Fig. 1). Based on the ROC curves, the cut-off value of TPS for PD-L1 positivity was estimated as “1”. Patients with PD-L1-positive tumors (TPS ≥ 1) seemed to show a worse prognosis, but the difference did not reach a statistical significance (Fig. 2).

### RFS and OS according to PD-L1 status after stratification by NLR status

**Figure 3:**
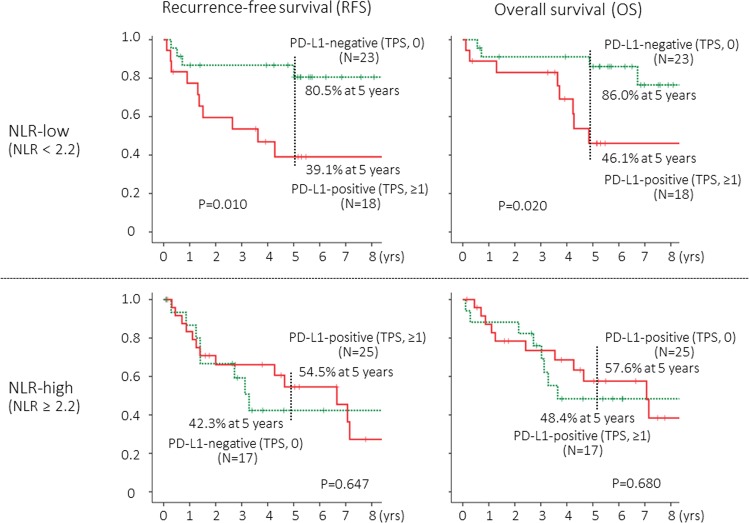
Recurrence-free survival (PFS) and overall survival (OS) curves according to tumor proportion score (TPS) for tumoral PD-L1 expression status among patients with low neutrophil-to-lymphocyte ratio (NLR, less than 2.2) or among high NLR (2.2 or higher) patients. The cut-off value for NLR and TPS were 2.2 and 1, respectively.

Among NLR-low (NLR < 2.2) patients, the prognostic impact on of PD-L1 status was significant (*P* = 0.010 for RFS and *P* = 0.020 for OS) (Fig. 3, upper). Among NLR-high (NLR ≥ 2.2) patients however, the prognostic impact disappeared (Fig. 3, lower).

### Prognostic impact of PD-L1 expression status in combination with NLR status

**Figure 4:**
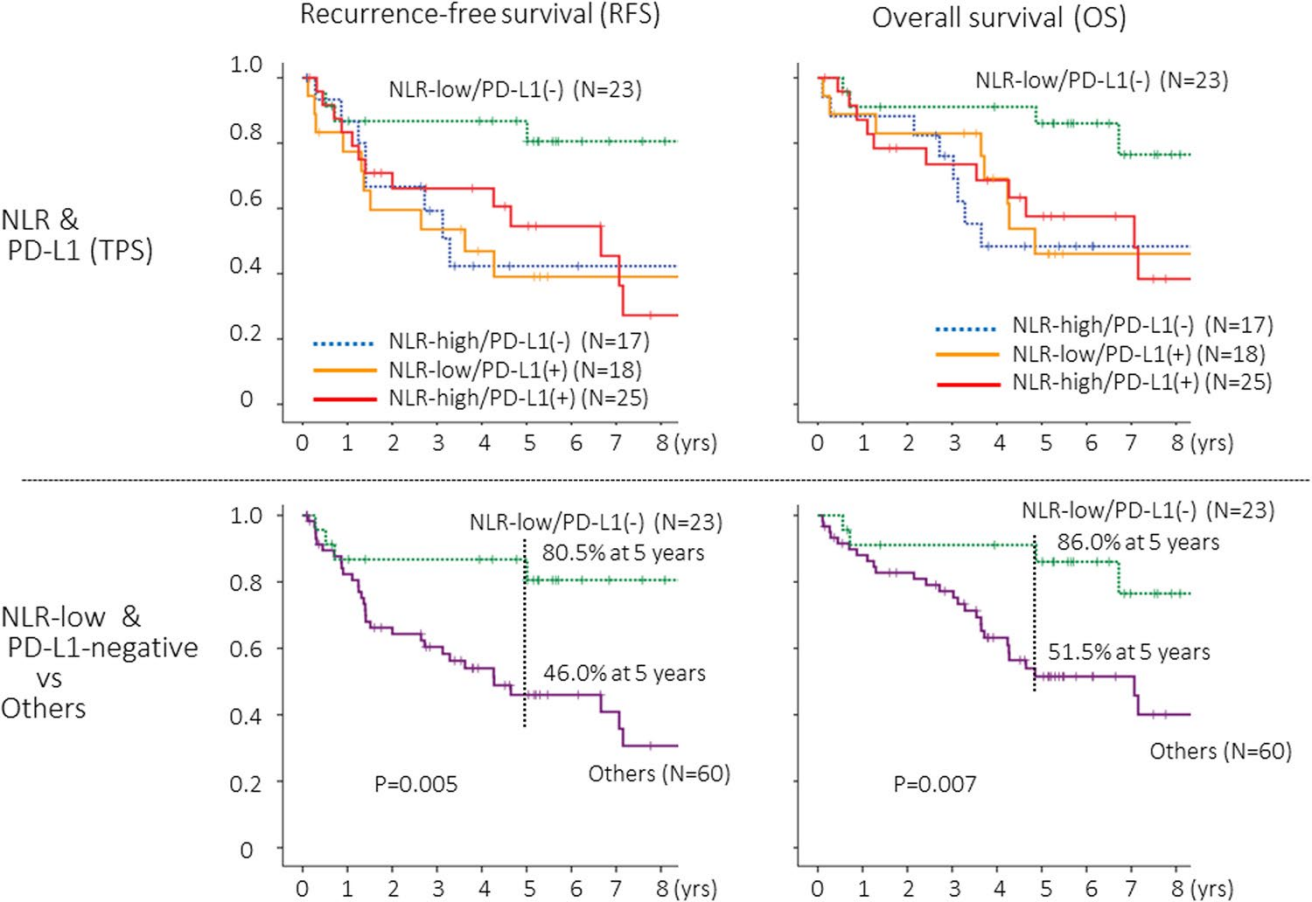
Recurrence-free survival (PFS) and overall survival (OS) curves according to tumor proportion score (TPS) for tumoral PD-L1 expression status in combination with status of neutrophil-to-lymphocyte ratio (NLR). The cut-off value for NLR and TPS were 2.2 and 1, respectively.

When NLR status and tumoral PD-L1 status were combined, “NLR-low (<2.2) and PD-L1-negative (TPS, 0)” patients showed the most favorable prognosis (Fig. 4, upper), and the difference was highly significant (Fig. 4, lower). Multivariate analyses in which age, sex, pathologic stage (stage IA or IB), mode of surgery (sub-lobar resection or lobectomy), adjuvant chemotherapy, and “NLR-low and PD-L1-negative” were included as variables showed that the “NLR-low and PD-L1-negative” was a significant and independent factor to predict a favorable RFS and OS (Table 2).

**Table 2:** Univariable and Multivariable Cox model of prognostic factors for recurrence-free survival and overall survival.

	Recurrence-free survival						Overall survival					
	Univariable analysis			Multivariable analysis			Univariable analysis			Multivariable analysis		
	HR	95% CI	*P-*	HR	95% CI	*P-*	HR	95%CI	*P-*	HR	95%CI	*P-*
Age (per 1-year increase)	1.016	0.972–1.063	0.476				1.031	0.981–1.083	0.229			
Sex, female (vs male)	0.791	0.307–2.037	0.791				0.777	0.272–2.218	0.637			
Smoking status, current smoker (vs never smoker)	1.049	0.543–2.027	0.887				1.058	0.526–2.129	0.874			
Pathologic stage IB (vs IA)	0.940	0.488–1.814	0.855				1.040	0.518–2.089	0.911			
Mode of lung resection, sub-lobar resection (vs lobectomy)	1.967	0.936–4.134	0.074				1.456	0.622–3.405	0.386			
Adjuvant chemotherapy, performed (vs not performed)	0.397	0.121–1.299	0.127				0.450	0.137–1.483	0.189			
**NLR-low/PD-L1(-) vs others**												
PD-L1(-): TPS = 0	0.237	0.083–0.674	0.007	0.239	0.082–0.693	0.008	0.260	0.091–0.745	0.012	0.276	0.094–0.808	0.019
PD-L1(-): TPS, 0–4	0.358	0.155–0.827	0.016	0.365	0.154–0.855	0.022	0.401	0.171–0.940	0.036	0.426	0.178–0.969	0.045
PD-L1(-): TPS, 0–9	0.396	0.179–0.878	0.023	0.392	0.173–0.888	0.025	0.378	0.161–0.883	0.025	0.390	0.164–0.928	0.033
PD-L1(-): TPS, 0–49	0.430	0.206–0.899	0.025	0.437	0.206–0.929	0.031	0.349	0.156–0.784	0.011	0.359	0.157–0.821	0.015

HR, hazard ratio; CI, confidence interval.

NLR, neutrophil-to-lymphocyte ratio.

TPS, tumor proportion score.

### Exploratory analyses at other TPS cut-off values

Exploratory analyses were performed at other cut-off values of TPS (5, 10, 50) that had been employed in previous clinical trials^7^, which showed similar results (Figs. S.1, S.2a, S.2b, S.3, and Table 2).

## Discussion

The current study first demonstrated that the prognostic impact of PD-L1 expression on TCs might be influenced according to NLR, and that the “NLR-low and PD-L1-negative” was significantly associated with a favorable prognosis in completely resected p-stage I squamous cell carcinoma of the lung.

PD-L1 expression status on TCs, as represented as TPS, is generally recognized as a potential biomarker to predict the efficacy of antibodies against PD-1 and PD-L1 for advanced NSCLC^4,6,7^, as its predictive values have been examined and indicated in many clinical studies. In clinical practice, for patients with advanced NSCLC with high PD-L1 expression (TPS ≥ 50), single agent first-line treatment with pembrolizumab, an anti-PD-1 antibody, is recommended as the standard treatment of care^15^. However, the prognostic significance of tumoral PD-L1 expression status for early-resectable NSCLC remains controversial, whereas a number of studies have been reported. In a recent meta-analysis of 38 studies^8^, positive PD-L1 expression on TCs was associated with worse OS (HR, 1.40 [95% CI, 1.20–1.69]) and RFS (HR, 1.67 [95% CI, 1.22–2.29]) overall, but conflicting results were indicated in some studies included in the meta-analysis. PD-L1 positivity was associated with a favorable prognosis in 11 (31.4%) of 35 studies for OS and in 2 (20.0%) of 10 studies for RFS, respectively^8^. These conflicting results may be caused by retrospective nature, relatively small number of patients, and heterogeneous patient characteristics. In addition, as indicated in the present study, the prognostic impact of PD-L1 expression on TCs may be influenced by the status of cancer immunity activity as well as by several factors associated in cancer immunity^6^.

Cancer immunity prevents development and progression of malignant tumor, which comprise a series of steps from release of neo-antigen from TCs and activation of CTLs through killing of target TCs by activated CTLs^5^. A number of molecules to upregulate or downregulate cancer immunity are involved at each step. PD-L1 is a potent negative regulator at the final step of cancer immunity. PD-L1 expressed on TCs binds to PD-1 on CTLs and downregulate immune function of activated CTLs. Accordingly, when cancer immunity is not activated prior to the final step of killing TCs by CTLs, TCs may survive regardless of PD-L1 expression. When cancer immunity is activated, TCs expressing no PD-L1 may be killed by activated CTLs and only TCs expressing PD-L1 in response to immune attack by CTLs may survive cancer immunity. The NLR can be a potential indicator of immune status as well as that of systemic inflammation^12–14,16^. Several clinical studies showed that low NLR was associated with a favorable prognosis in patients treated with inhibitors of PD-1/PD-L1^13,14,17–21^. Accordingly, the prognostic impact of PD-L1 expression status may be influenced by the status of NLR. In fact, “NLR-low and tumoral PD-L1-negative” patients showed a favorable prognosis in the present study.

The present exploratory study indicated that the prognostic impact of PD-L1 expression on TCs might be influenced by the status of NLR, but this study had several limitations due to a variety of weakness. First, only 83 patients were included in the study, and its relatively small number of patients may not provide an enough statistical power to detect some difference. For example, PD-L1-positivity (TPS ≥ 1) seemed to be correlated with a worse prognosis (Fig. 2), but the difference did not reach a statistical significance (P = 0.056) in this study. In a future prospective study, the sample size shall be calculated to detect an expected difference at the time of planning. Second, this study was a retrospective single-institutional study. Finally, patients with p-stage I squamous cell carcinoma were eligible, but there still remain some heterogeneity in patient characteristics. To draw definitive results, larger-scale clinical studies should be conducted.

## Material and Methods

### Patients

Patients with p-stage I squamous cell carcinoma of the lung, who received complete resection without induction treatment prior to surgery at our institute from 2003 through 2012 were retrospectively reviewed. Patients who did not provide written informed consent for this study were excluded. Patients were ineligible when adequate primary tumor samples for immunohistochemistry (IHC) were not available, and a total of 83 patients were finally included in this study (Table 3).

**Table 3:** Characteristics of patients.

Characteristic		All patients (n = 83)
Age, median (years)		74.0 (45–85)
Sex	Female	13 (15.7%)
	Male	70 (84.3%)
Smoking status	Never	1 (1.2%)
	Former	39 (47.0%)
	Current	43 (51.8%)
Brinkman index, median (pack-year)		56.0 (0–162)
Cell differentiation	Well/Moderately/Poorly	18 (21.7%)
	Moderately	54 (65.1%)
	Poorly	11 (13.3%)
Pathologic stage	IA	41 (49.4%)
	(IA1/IA2/IA3)	(1/15/25)
	IB	42 (50.6%)
Mode of lung resection	Sub-lobar resection	18 (21.7%)
	Lobectomy	65 (78.3%)
Adjuvant chemotherapy	Performed	11(13.3%)
	(UFT/Platimun-based)	(4/7)
NLR, median (range)		2.2 (0.9–7.5)
PD-L1 expression (TPS)	0	40 (48.2%)
	≥1%	43 (51.8%)
	≥5%	31 (37.3%)
	≥10%	25 (30.1%)
	≥50%	14 (16.9%)

Data represented as absolute counts (%) or median (range).

NLR, neutrophil-to-lymphocyte ratio.

TPS, tumor proportion score.

UFT, tegafur and uracil.

Whole-body computed tomography (CT), brain magnetic resonance imaging (MRI) and bone scan were performed preoperatively. P-stage was re-evaluated according to the current tumor, node, metastases (TNM) classification (IUCC TNM staging system, 8th edition)^22^. Lobectomy was principally performed, but sub-lobar resection was actually performed in 18 patients (21.7%) who were not fit for lobectomy. No postoperative adjuvant treatment was principally prescribed, but eleven patients (13.3%) who were enrolled in clinical trials received assigned adjuvant chemotherapy after surgery (Table 3). Lymphocyte count and neutrophil count were obtained from the routine preoperative blood test. The NLR was calculated by dividing the neutrophil count by the lymphocyte count. Each blood for the NLR was sampled within 7 days prior to surgery. The institutional review board of the University of Occupational and Environmental Health, Japan approved the present study. A written informed consent was obtained from each patient. All experiments were performed in accordance with relevant guidelines and regulations.

### Evaluation of tumoral PD-L1 expression

PD-L1 expression on TCs was evaluated with IHC. Serial 4μm-sections were cut from each formalin-fixed and paraffin-embedded primary tumor specimen that had been taken at surgery. Sections were served for hematoxylin and eosin (HE) staining and IHC as described previously^9,23^. Briefly, after antigen retrieval by heating in 1 mM EDTA (pH 8.0) at 98 °C for 15 minutes, sections were incubated with a rabbit anti-PD-L1 monoclonal antibody (clone E1L3N, Cell Signaling Technology Japan, Tokyo) diluted at 1:200 for 60 minutes. Thereafter, sections were incubated with the SignalStain Boost IHC Detection Reagent HRP Rabbit (Cell Signaling Technology Japan).

Each slide was independently evaluated by two of the investigators (T.K. and A.H.) without knowledge of any clinical data. The percentage of tumor cells with membrane staining for PD-L1 (TPS) was recorded. When a discrepancy was found between the two investigators, the slide was reviewed via their simultaneous examination using a double-headed microscope to achieve a consensus.

### Statistical analysis

Proportions of categorical data were compared by the chi-square test. Continuous data were compared using a non-parametric test (Mann-Whitney U-test). Spearman’s rank correlation coefficients (two-sided) were used to evaluate correlations between NLR and TPS. ROC curve analyses were performed to determine the optimal cut-off values of NLR and TPS.

The RFS was defined as the time from surgery to tumor recurrence or death from any cause. The OS was defined as the time from surgery to death of any cause. A telephone follow-up would be made if the patient did not come to our clinic for a routine follow-up. The Kaplan-Meier method was used to estimate probability of survival, and survival differences were analyzed by the log-rank test. To identify independent prognostic factors, a multivariable analysis was performed using a Cox proportional hazards regression model. The HR and 95% CI were calculated for each variable.

Differences were considered to be statistically significant for p values < 0.05. All statistical analyses were performed with the SPSS version 21 software (IBM Corp., Armonk, NY).

## Conclusions

The prognostic impact of PD-L1 expression on TCs was distinct according to NLR in completely resected p-stage I squamous cell carcinoma of the lung. PD-L1-positivity on TCs was associated with a poor prognosis among NLR-low patients, but it provided no prognostic impact among NLR-high patients. “NLR-low and tumoral PD-L1-negative” patients showed a favorable prognosis.

## Supplementary information

Supplementary  Information
