# Supplementary material for: Prognostic impact of PD-L1 expression in correlation with neutrophil-to-lymphocyte ratio in squamous cell carcinoma of the lung
Source: Sci Rep. 2020 Jan 27;10:1243. doi: 10.1038/s41598-019-57321-x (PMC6985257; doi:10.1038/s41598-019-57321-x)
Supplement: Supplementary file 1 — Supplementary Information [file 41598_2019_57321_MOESM1_ESM.pdf]

Prognostic impact of PD-L1 expression in correlation with neutrophil-to-lymphocyte ratio in squamous cell carcinoma of the lung

Yuko Tashima*, Taiji Kuwata*, Kazue Yoneda, Ayako Hirai, Masataka Mori, Masatoshi Kanayama, Naoko Imanishi, Koji Kuroda, Yoshinobu Ichiki, Fumihiro Tanaka

Second Department of Surgery (Chest Surgery), University of Occupational and Environmental Health, Kitakyushu, Japan

*The two authors equally contributed to the study

Legends of supplementary figures

Figure S.1

Recurrence-free survival (PFS) and overall survival (OS) curves according to tumor proportion score (TPS) at each cut-off (5, 10, 50) for tumoral PD-L1 expression status.

Figure S.2a

Recurrence-free survival (PFS) and overall survival (OS) curves according to tumor proportion score (TPS) at each cut-off (5, 10, 50) for tumoral PD-L1 expression status among patients with low neutrophil-to-lymphocyte ratio (NLR, less than 2.2)

Figure S.2b

Recurrence-free survival (PFS) and overall survival (OS) curves according to tumor proportion score (TPS) at each cut-off (5, 10, 50) for tumoral PD-L1 expression status among patients with high neutrophil-to-lymphocyte ratio (NLR, 2.2 or higher)

Figure S.3

Recurrence-free survival (PFS) and overall survival (OS) curves according to tumor proportion score (TPS) at each cut-off (5, 10, 50) for tumoral PD-L1 expression status in combination with status of neutrophil-to-lymphocyte ratio (NLR).

Figure S.1

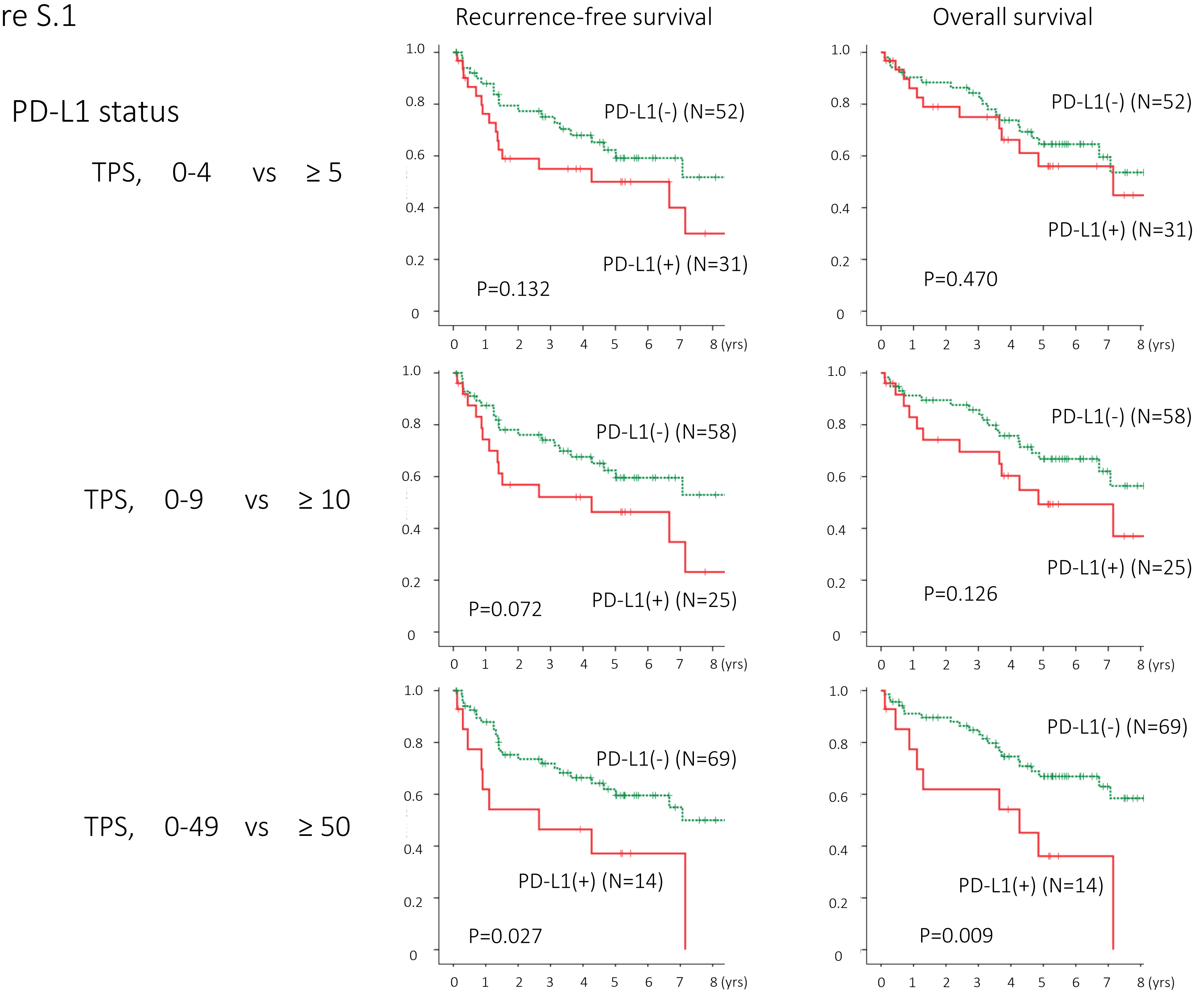
Recurrence-free survival
Overall survival
PD-L1 status
TPS, 0-4 vs ≥ 5
PD-L1(-) (N=52)
PD-L1(+) (N=31)
P=0.132
P=0.470
TPS, 0-9 vs ≥ 10
PD-L1(-) (N=58)
PD-L1(+) (N=25)
P=0.072
P=0.126
TPS, 0-49 vs ≥ 50
PD-L1(-) (N=69)
PD-L1(+) (N=14)
P=0.027
P=0.009
1.0
0.8
0.6
0.4
0.2
0
0 1 2 3 4 5 6 7 8 (yrs)

Figure S.2a

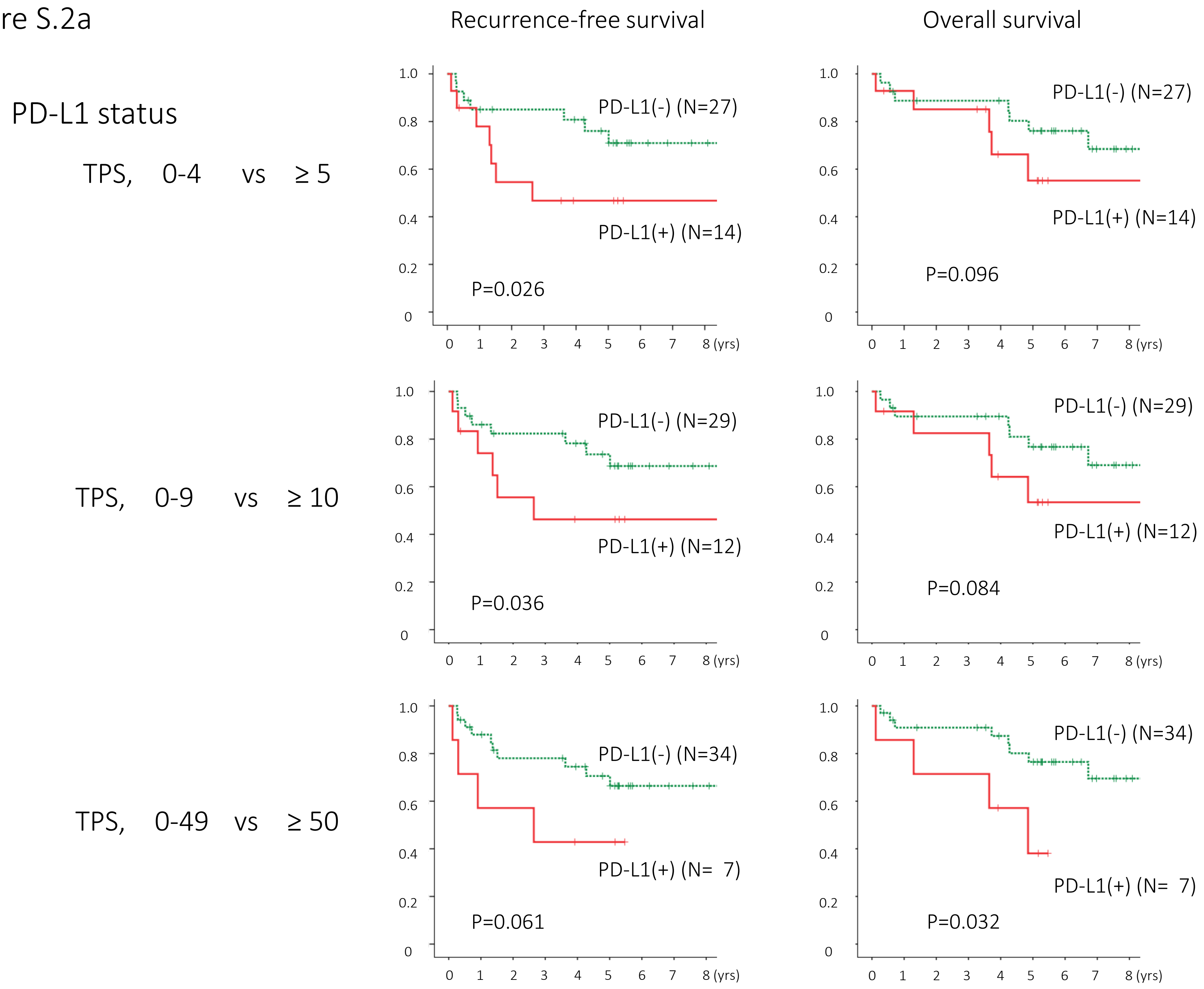

Recurrence-free survival
Overall survival
PD-L1 status
TPS, 0-4 vs ≥ 5
PD-L1(-) (N=27)
PD-L1(+) (N=14)
P=0.026
P=0.096
TPS, 0-9 vs ≥ 10
PD-L1(-) (N=29)
PD-L1(+) (N=12)
P=0.036
P=0.084
TPS, 0-49 vs ≥ 50
PD-L1(-) (N=34)
PD-L1(+) (N= 7)
P=0.061
P=0.032
1.0
0.8
0.6
0.4
0.2
0
0 1 2 3 4 5 6 7 8 (yrs)

Figure S.2b

PD-L1 status

TPS, 0-4 vs ≥ 5

TPS, 0-9 vs ≥ 10

TPS, 0-49 vs ≥ 50

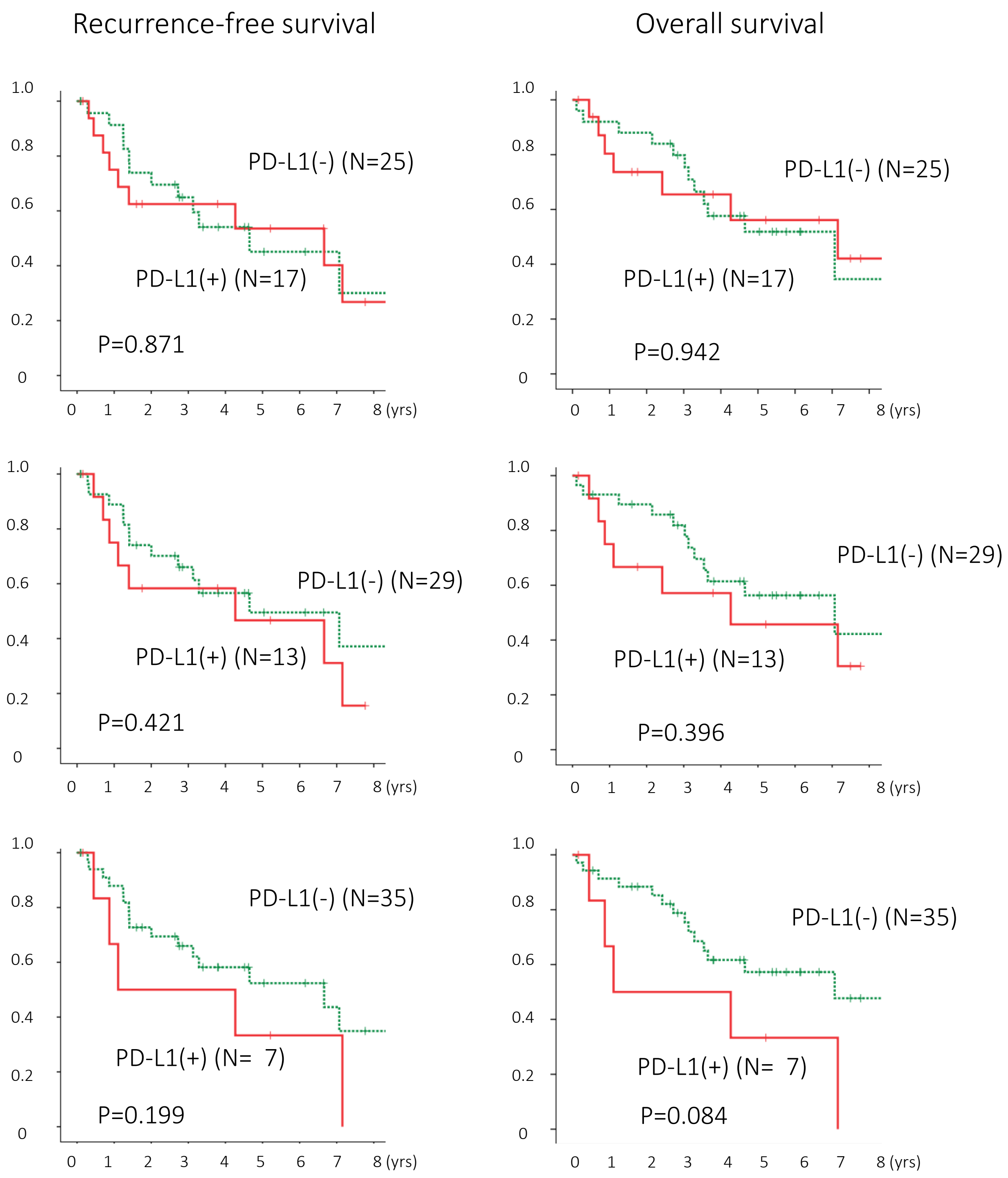

Figure S.3

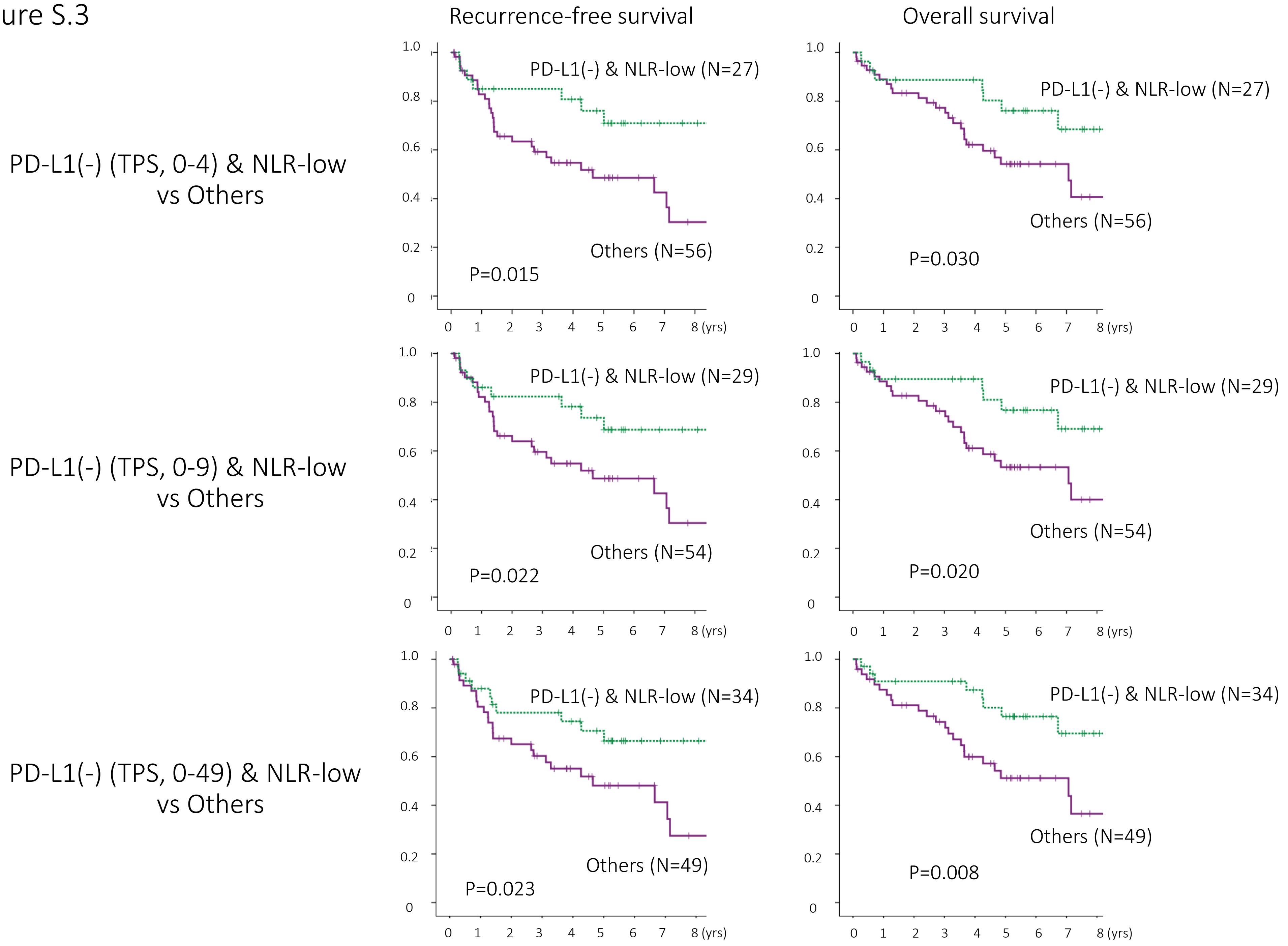
Recurrence-free survival
Overall survival
PD-L1(-) (TPS, 0-4) & NLR-low vs Others
PD-L1(-) & NLR-low (N=27)
Others (N=56)
P=0.015
P=0.030
PD-L1(-) (TPS, 0-9) & NLR-low vs Others
PD-L1(-) & NLR-low (N=29)
Others (N=54)
P=0.022
P=0.020
PD-L1(-) (TPS, 0-49) & NLR-low vs Others
PD-L1(-) & NLR-low (N=34)
Others (N=49)
P=0.023
P=0.008
1.0
0.8
0.6
0.4
0.2
0
0 1 2 3 4 5 6 7 8 (yrs)
